# RACK1 promotes maintenance of morphine-associated memory via activation of an ERK-CREB dependent pathway in hippocampus

**DOI:** 10.1038/srep20183

**Published:** 2016-02-02

**Authors:** Litao Liu, Jiejun Zhu, Liming Zhou, Lihong Wan

**Affiliations:** 1 Department of Pharmacology, West China School of Preclinical and Forensic Medicine, Sichuan University, Chengdu, Sichuan 610041, P.R. China; 2 Sichuan University “985 project - Science and Technology Innovation Platform for Novel Drug Development”, Sichuan University, Chengdu, Sichuan 610041, P.R. China

## Abstract

Existence of long-term drug-associated memories may be a crucial factor in drug cravings and relapse. RACK1 plays a critical role in morphine-induced reward. In the present study, we used conditioned place preference (CPP) to assess the acquisition and maintenance of morphine conditioned place preference memory. The hippocampal protein level of RACK1 and synaptic quantitation were evaluated by Western blotting, immunohistochemistry and electron microscopy, respectively. Additionally, shRACK1 (shGnb2l1) was used to silence RACK1 *in vivo* to evaluate the role and the underlying mechanism of RACK1 in maintenance of morphine CPP memory. We found that morphine induced CPP was maintained for at least 7 days after the last morphine treatment, which indicated a positive correlation with hippocampal RACK1 level, and was accompanied simultaneously by increases in the synapse density and hippocampal expression of synaptophysin (SYP), phosphorylation of extracellular signal-regulated kinase1/2 (pERK1/2) and the phosphorylation of cyclic adenosine monophosphate response element-binding (pCREB). ShGnb2l1 icv injection significantly suppressed the expression of all above proteins, decreased the synapse density in the hippocampus and attenuated the acquisition and maintenance of morphine CPP. Our present study highlights that RACK1 plays an important role in the maintenance of morphine CPP, likely via activation of ERK-CREB pathway in hippocampus.

Opioids are the most commonly used analgesics in the treatment of moderate-to-severe pain. However, prolonged or repeated administration of opioids may lead to addiction^1^. Morphine is a representative opioid with high abuse liability. Currently, one of the main challenges in the treatment of morphine addiction is the high relapse rate of morphine use, even after a long-term abstinence^2^,^3^. Cues associated with prior drug use can reactive memories associated with prior drug use and result in relapse^4^. Thus, the existence of long-term drug-associated memories is believed to be a crucial factor in the onset of drug craving and relapse^5^. The conditioned-place preference (CPP) paradigm is a widely used animal model to investigate the mechanisms of drug memory. Acquisition of CPP represents the initial phase of drug use when memories associated with the reinforcing effects of a drug are formed.

The hippocampus plays a central role in the consolidation of information from short-term memory to long-term memory^6^. Accumulating evidence suggests that synaptic plasticity occurs in hippocampus resulting in an alteration of the transfer of information throughout the brain’s rewardsystem^7^. For example, morphine consumption could significantly increase the synaptic numbers, postsynaptic density and curvature of the synaptic interface in rat’s hippocampus^8^. Further, the increased basal synaptic transmission is associated with morphine induced CPP^9^.

The receptor for activated protein kinase C 1 (RACK1) has been identified as an important inhibitory scaffolding protein involved in the regulation of diverse cellular activities^10^ including channel activity^11^, cell apoptosis^12^ and synaptic plasticity^13^. Previous reports have revealed that RACK1 activates the transcription of BDNF by binding to the promoter IV region of BDNF gene to contribute to synaptic plasticity, learning, and memory^14^. In our previous investigations, we have also found that shRACK1 (shGnb2l1) icv injection significantly attenuated morphine conditioned CPP via down-regulation of BDNF-CREB signaling in the hippocampus^15^,^16^. However, there is little information to-date regarding the precise role and mechanism of RACK1 in the hippocampus, particularly its role in maintaining morphine conditioned place preference memory.

Extracellular signal-regulated kinase (ERK1/2), a member of the mitogen-activated protein kinase (MAPK) pathway, is a main player in the main intracellular pathway activated by BDNF-TrkB signaling^17^. A growing body of evidence supports that ERK1/2 and its phosphorylated protein (pERK1/2) are involved in the pathophysiology of synaptic plasticity, memory formation and drug addiction^18^,^19^,^20^. Treatment with an ERK inhibitor could attenuate ERK1/2 phosphorylation and prevent the expression of morphine-induced CPP^19^. The downstream regulator of the ERK cascade, cyclic-AMP response element binding protein (CREB), has also been demonstrably involved in the development of drug dependence and addiction. Context-dependent administration of morphine could increase CREB mRNA and protein level^15^ and CREB phosphorylation in the hippocampus and cortex^21^. Specially, CREB is an important transcription factor that modulates the transcription of many genes, including the presynaptic protein synaptophysin (SYP), by enrichment of phosphorylated CREB on promoters of this gene^22^. Together, these data suggest that RACK1 in the hippocampus may lead to changes in synaptic numbers and SYP expression mediating via the ERK-CREB signaling pathway and may be crucial for morphine conditioned CPP maintenance.

In the present study, we first determined whether the systemic administration of morphine (10 mg/kg) in mice would induce persistent CPP and enhanced RACK1 or SYP expressions in the hippocampus 7 days after cessation of morphine. Subsequently, we used a small hairpin RNA shRACK1 (shGnb2l1) to silence RACK1 in the brains of mice in a model of morphine addiction to further explore the relationship of RACK1 and morphine conditioned place preference memory.

## Results

### Persistent morphine conditioned place preference memory was positively correlated with RACK1 in hippocampus

**Figure 1:**
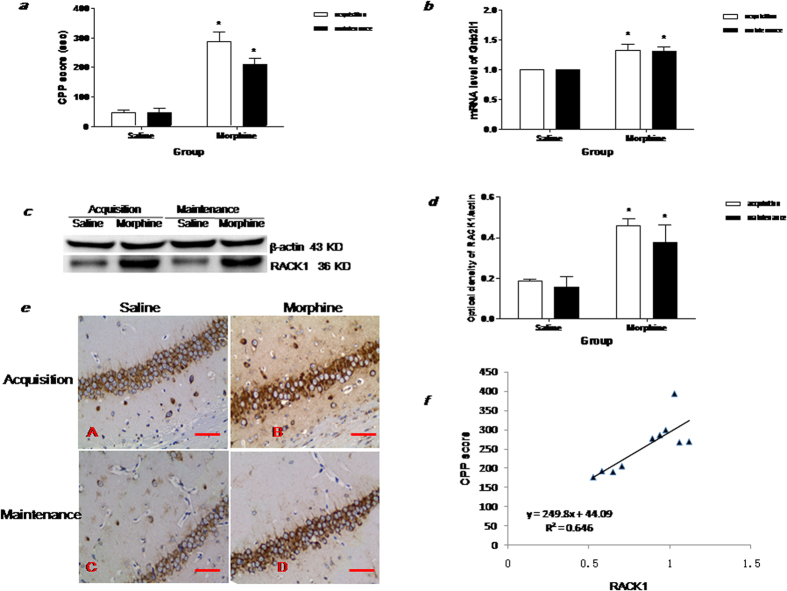
Repeated morphine treatment induced persistent conditioned place preference memory and up-regulated RACK1 levels in hippocampus. (**a**) Morphine induced conditioned place preference was conducted on day 14 and 21. CPP scores = time spent in the drug-paired compartment_test_-time spent in the drug-paired compartment_pretest_. CPP scores in mice are given as mean ± SEM under the different conditions. The white column shows the CPP scores in acquisition (Test in day 14). The black column shows the CPP scores in maintenance (Test in day 21). ^*^p < 0.05 compared with saline group (n = 10); (**b**) The mRNA level of RACK1 (Gnb2l1) in hippocampus (HP) was analyzed with real-time PCR,^*^p < 0.05 compared with saline group (n = 5); (**c**) Representative immunoblots showing the molecular weights of the proteins used to quantify level of RACK1 (36 kDa) in the HP. Signals were normalized to β-actin (43 kDa); (**d**) Comparison of the intensity of the RACK1/actin band between the experimental groups, ^*^p < 0.05 compared with saline group (n = 5); (**e**) Location of RACK1 protein in CA1 of HP was measured by IHC. The brown cells represent the RACK1 positive cells in the DAB immunostaining. (**a**) Saline treatment mice in acquisition; (**b**) Morphine treatment mice in acquisition; (**c**) Saline treatment mice in maintenance; (**d**) Morphine treatment mice in maintenance; The scale bar = 0.5 mm; (**f**) Positive correlation of RACK1 protein levels with CPP scores (r^2^ = 0.7418, p < 0.05).

Statistical analysis (two-way ANOVA) revealed a significant difference between saline- and 10 mg/kg morphine-treated mice (F_(1,10)_ = 125.5, p < 0.001) though no significant effects of time were detected (F_(1,10)_ = 2.579, p = 0.139). The Bonferroni post-hoc test demonstrated that the CPP score of morphine-conditioned mice was significantly higher than that of saline-treated mice (t = 8.071, p < 0.001) on day 14 of CPP acquisition. Moreover, in another experimental group, the mice with established morphine conditioned CPP were returned to their home cage without any further treatment for 7 days (days 14–20). On day 21, the morphine treated mice continued to demonstrate a significant preference for the morphine paired compartment (Fig. 1a) compared with the saline-treated mice (t = 5.479, P < 0.001), suggesting that the morphine-induced CPP was maintained for at least 7 days after the last morphine treatment.

Messenger RNA and protein level of RACK1 in the hippocampus was measured by real-time RT-PCR, western blotting and immunohistochemistry on day 14 (acquisition phase) and 21 (maintenance phase) of the morphine time course. Statistical analysis (two-way ANOVA) revealed a significant difference between saline- and 10 mg/kg morphine-treated mice (mRNA: F(1, 4) = 19.66, p = 0.01; Protein: F(1, 4) = 13.61, p = 0.02) though no significant effects of time were detected (F(1, 4) = 0.0038, p = 0.95; Protein: F(1, 4) = 2.609, p = 0.18). The Bonferroni post-hoc test demonstrated that the mRNA and protein level of RACK1 in hippocampus were significantly increased on day 14 (acquisition phase) in morphine-treated mice (mRNA: t = 3.531, P < 0.05; Protein: t = 3.618, P < 0.05). Additionally, we found that increases of mRNA and protein level of RACK1 in the hippocampus were maintained at least 7 days after the acquisition of morphine CPP (maintenance phase) (mRNA: t = 3.455, P < 0.05; Protein: t = 2.963, P < 0.05) (Fig. 1b–d). The immunohistochemistry results also indicated that pyramidal neurons (Pyr) in the CA1 of the hippocampus were strongly immunoreactive for the RACK1 antibody during both the acquisition and maintenance phase (Fig. 1e). More importantly, a correlation analysis between the protein expression of RACK1 in the hippocampus and morphine induced CPP score revealed a significant positive correlation between these two parameters in the morphine group (Fig. 1f) (r2 = 0.646, P < 0.001), with higher RACK1 protein expression predicting a higher degree of morphine conditioned place preference memory. Moreover, hippocampal RACK1 mRNA and protein expressions are significantly increased in conditioned paired chronic morphine exposure mice, but not in conditioned unpaired chronic morphine exposure mice (Fig. S1).

### Synaptogenesisis involved in the maintenance of morphine conditioned place preference memory

**Figure 2:**
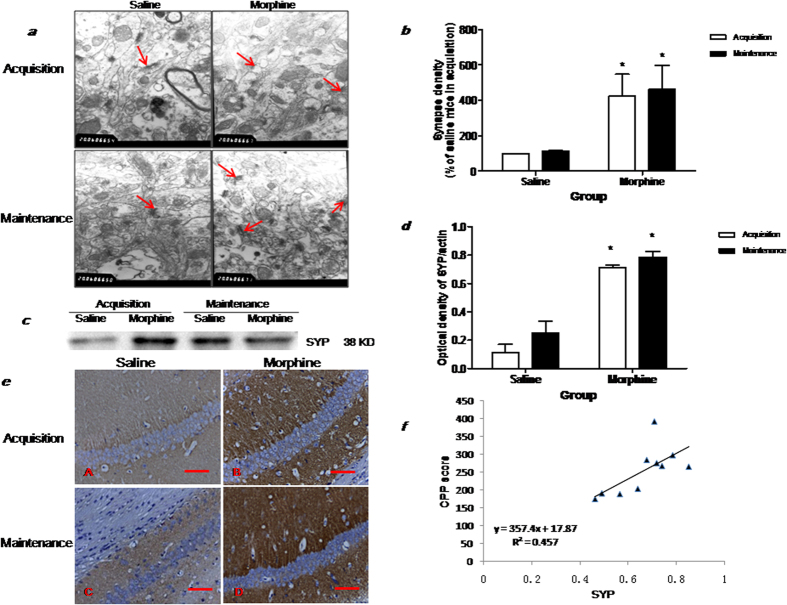
Change of synaptogenesis after chronic morphine administration and withdrawal 7 days. (**a**) Ultrastructural changes in CA1 of HP were observed by TEM, the arrow indicates a synapse. Scale bar = 20 μM; (**b**) Quantification of synapse density in CA1; data (mean ± SEM) are expressed as percent of synapse density normalized to the appropriate control (the group that received saline injections in training phase), ^*^p < 0.05 compared with saline group (n = 5); (**c**) Representative immunoblot showing the molecular weights of the proteins used to quantify level of SYP (38 kDa) in HP. Signals were normalized to β-actin (43 kDa); (**d**) Comparison of the intensity of the SYP/actin band between the experimental groups,^*^p < 0.05 compared with saline group (n = 5); (**e**) Location of SYP protein in CA1 of HP was measured by IHC. The brown cells represent the SYP positive cells in DAB immunostaining. (**a**) Saline treatment mice in acquisition; (**b**) Morphine treatment mice in acquisition; (**c**) Saline treatment mice in maintenance; (**d**) Morphine treatment mice in maintenance; The scale bar = 0.5 mm; (**f**) Positive correlation of SYP protein level with CPP scores (r^2^ = 0.5285, p < 0.05).

To explore the relationship of hippocampal synaptogenesis and the maintenance of morphine conditioned place preference memory, synaptic density and synaptophysin expression (SYP) were used to evaluate synaptogenesis^23^. Using electron microscopy^24^, we measured the synaptic density in the CA1 of the hippocampus (Fig. 2a). By calculating the ratio of the synapse density, which is normalized to the appropriate control (in this case a group that received saline injections during the acquisition phase), we found that synaptic density was notably increased during both acquisition (t = 2.505, P < 0.05) and maintenance phases (t = 2.703, P < 0.05) (Fig. 2b). Western blotting additionally showed that both phases were concordant with significantly increased SYP protein expression in the hippocampus, as compared to saline treated mice (F(1, 4) = 253.9, p < 0.0001, Fig. 2c,d). Further immunohistochemical analysis showed that morphine treated mice exhibited higher SYP expression in the CA1 region (Fig. 2e), similar to that observed in western blotting analysis. These data collectively indicated an increase in synaptogenesis occurring after morphine treatment.

A significant positive correlation was observed between the protein expression of SYP in the hippocampus and morphine induced CPP score (similar to RACK1 and morphine induced CPP score) (Fig. 2f) (r2 = 0.457, P < 0.001), with higher SYP protein expression predicting a higher degree of morphine conditioned place preference memory. Moreover, synaptogenesis is also significantly increased in conditioned paired chronic morphine exposure mice, but not in conditioned unpaired chronic morphine exposure mice (Fig. S1).

### shGnb2l1 silenced the expression of RACK1 and impaired the acquisition and maintenance of morphine conditioned place preference memory by suppressing synaptic plasticity

To obtain more information concerning the role of RACK1 in persistent morphine conditioned place preference memory, we designed two separate experiments (acquisition experiment and maintenance experiment), in both of these experiments, mice were divided into four groups: saline + shNC, saline + shGnb2l1, morphine + shNC and morphine + shGnb2l1.

**Figure 3:**
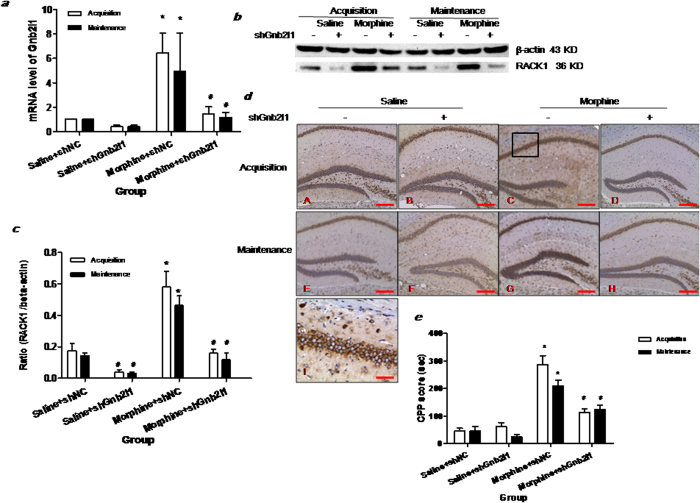
Effect of shGnb2l1 icv injection during acquisition and maintenance of morphine-induced reward. (**a**) shGnb2l1 icv injection decreased the mRNA level of RACK1 (Gnb2l1) in HP (HP) (n = 5); (**b**) shGnb2l1 icv injection decreased the protein expression of RACK1 in HP; (**c**) Comparison of the intensity of the RACK1/actin band between the experimental groups; (**d**) shGnb2l1 icv injection significantly decreased the expression of RACK1 protein in pyramidal neurons (Pyr) in the CA1 of HP in the acquisition and maintenance experiment. The brown cells represent the RACK1 positive cells in the DAB immunostaining. (**a,b**) saline + shNC mouse; (**b,f**) saline + shGnb2l1 mouse; (**c,g**) morphine + shNC mouse; (**d,h**) morphine + shGnb2l1 mouse; (**i**) Enlarged, from (**c**) shows that pyramidal neurons (Pyr) in the CA1 of the HP are strongly positive for RACK1; (**a–h**) The scale bar = 2 mm (**i**) The scale bar = 0.5 mm. (**e**) shGnb2l1 icv injection decreased the CPP score. CPP scores in mice are given as mean ± SEM under the different conditions. The white column shows the CPP scores in acquisition (Test in day 14). The black column shows the CPP scores in maintenance (Test in day 21). ^*^p < 0.05 compared with saline + shNC group (n = 10); ^#^p < 0.05 compared with morphine + shNC group (n = 10).

First, we treated the saline mice with shGnb2l1 or shNC (the control shRNA plasmid) via i.c.v injection and evaluated the interference efficiency (IE) of shGnb2l1 in the hippocampus by realtime RT-PCR. Under Gnb211 interference, the level of RACK1 mRNA was significantly decreased in the hippocampus (IE = 60%, Fig. 3a). Further investigation using Western blotting and immunochemistry continually supported that shGnb2l1 i.c.v injection could significantly inhibit RACK1 protein levels in the hippocampus (Fig. 3b,c), especially in the CA1 region (Fig. 3d).

Statistical analysis (two-way ANOVA) revealed a significant difference between treatments (morphine + ShGnb211 versus morphine + ShNC) in mRNA and protein expression of RACK1 in the hippocampus (mRNA: F(3, 8) = 19.83, p < 0.001, Fig. 3a; protein: F(3, 8) = 19.83, p < 0.001, Fig. 3b,c) though no significant effects of time were detected (mRNA: F(1, 8) = 0.812, p = 0.3938, Fig. 3a; protein: F(1, 8) = 3.713, p = 0.09, Fig. 3b,c). Tukey’s post-hoc test demonstrated that morphine could increase RACK1 mRNA and protein levels in acquisition experiment (mRNA: q = 7.43, p < 0.05; protein: q = 8.293, p < 0.05) and in maintenance experiment (mRNA: q = 5.394, p < 0.05; protein: q = 6.551, p < 0.05), which is consistent with our previous study. When compared to the morphine + shNC group, the mRNA and protein expression of RACK1 in hippocampus of shGnb2l1-treated morphine mice was significantly lower in the acquisition experiment (mRNA: q = 6.802, p < 0.05; protein: q = 8.615, p < 0.05) as well as during the maintenance experiment (mRNA: q = 5.148, p < 0.05; protein: q = 7.112, p < 0.05). Similar results were observed in immunohistochemical analysis, as shown in Fig. 3d where the RACK1 protein in pyramidal neurons (Pyr) of the CA1 of hippocampus were strongly suppressed during the acquisition experiment and maintenance experiment.

Notably, parallel with the changes in RACK1 level, significant differences were detected between groups in CPP score (F(3, 20) = 68.91, p < 0.0001) though once again, no significant effects of time (F(1,20) = 3.841, p = 0.064, Fig. 3e) were found between the morphine + shNC and morphine + shGnb2l1 group. A post-hoc test specifically revealed a significant decrease in the CPP score of morphine + shGnb2l1 mice both in the acquisition experiment (q = 9.78, p < 0.05) and in the maintenance experiment (q = 4.871, p < 0.05) when compared to the morphine + shNC group.

**Figure 4:**
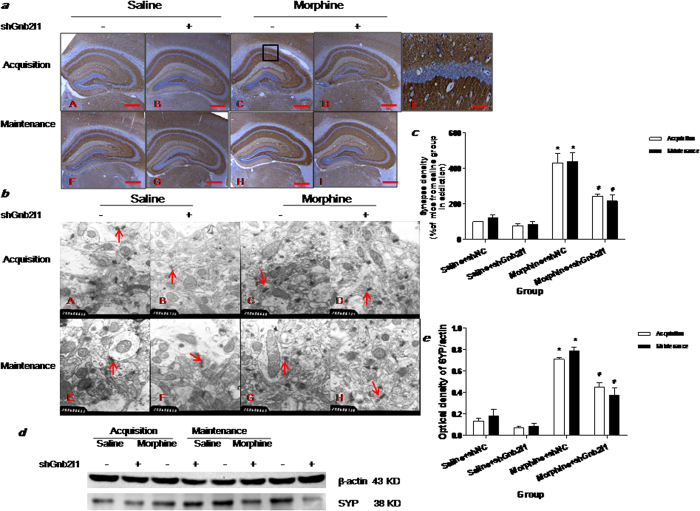
Effect of shGnb2l1 icv injection on synaptogenesis. (**a**) shGnb2l1icv injection significantly decreased the expression of SYP protein in the CA1 of HP in acquisition and maintenance. The brown cells represent the SYP positive cells in the DAB immunostaining. The scale bar = 2 mm. (**a,f**) saline + shNC mouse; (**b,g**) saline + shGnb2l1 mouse; (**c,h**) morphine + shNC mouse; (**d,i**) morphine + shGnb2l1 mouse; (**e**) Enlarged, from (**c**) shows that pyramidal neurons (Pyr) in the CA1 of the HP are strongly positive for SYP; (**a–d,f–i**) The scale bar = 2 mm; (**e**) The scale bar = 0.5 mm. (**b**) Ultrastructural changes in CA1 of HP were observed by TEM, the arrow indicates a selected synapse. (**a,e**) saline + shNC mouse; (**b,f**) saline + shGnb2l1 mouse; (**c,g**) morphine + shNC mouse; (**d,h**) morphine + shGnb2l1 mouse; Scale bar = 20 μM; (**c**) Quantification of synapse density in CA1; data (mean ± SEM) are expressed as percent of synapse density normalized to the appropriate control (saline + shNC in addiction experiment), ^*^p < 0.05 compared with saline + shNC group (n = 5); ^#^p < 0.05 compared with morphine + shNC group (n = 5); (**d**) shGnb2l1 icv injection decreased the protein expression of SYP in HP; (**e**) Comparison of the intensity of the SYP/actin band between the experimental groups, ^*^p < 0.05 compared with saline + shNC group (n = 5); ^#^p < 0.05 compared with morphine + shNC group (n = 5).

To further confirm the role of RACK1 in the regulation of hippocampal synaptogenesis, we examined whether the changes in synaptic density and synaptophysin expression (SYP) were observed after shGnb2l1 injection and whether they were accompanied by alterations of RACK1 and CPP. Immunohistochemistry showed that SYP expression level was notably decreased in the CA1 region in morphine + shGnb2l1 mice both in the acquisition experiment and in the maintenance experiment (Fig. 4a). As shown in Fig. 4b,c, two-way ANOVA and post hoc analysis showed that there was a significant difference among all groups (F(3,8) = 34.88, p < 0.0001, Fig. 4b,c) regarding synaptic density, which was markedly decreased after shGnb2l1 injection in the acquisition (q = 6.155, P < 0.05) and maintenance experiment (q = 7.321, P < 0.05). Western blotting also demonstrated that both in the acquisition and maintenance experiment, the expression of hippocampal SYP protein was notably inhibited in morphine + shGnb2l1 mice, as compared to morphine + shNC mice (F(3,8) = 137.2, p < 0.0001, Fig. 4d,e).

Taken together, these data suggested that shGnb2l1 silenced the expression of RACK1 and attenuated the acquisition and maintenance of morphine conditioned place preference memory at least for 7 days by suppressing hippocampal synaptogenesis.

### RACK1 contributes to the maintenance of morphine conditioned place preference memory by activation of ERK-CREB dependent pathway

Considering that the presynaptic protein SYP is a downstream target of the ERK/CREB pathway, we sought to elucidate the regulating mechanism of RACK1 on hippocampal synaptogenesis during the acquisition and maintenance experiment. To accomplish this we measured the protein expression of ERK1/2, pERK1/2, CREB, and pCREB by western blotting and immunohistochemistry.

**Figure 5:**
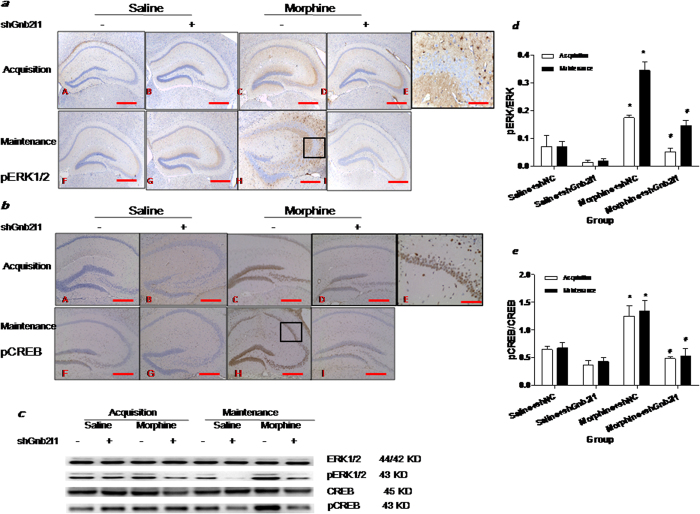
Effect of shGnb2l1 icv injection on ERK/CREB pathway. (**a**) shGnb2l1 icv injection significantly decreased the expression of pERK1/2 protein in the CA2 of HP in acquisition and maintenance. The brown cells represent the pERK1/2 positive cells. The scale bar = 0.5 mm. (**a,f**) saline + shNC mouse; (**b,g**) saline + shGnb2l1 mouse; (**c,h**) morphine + shNC mouse; (**d,i**) morphine + shGnb2l1 mouse; (**e**) Enlarged, from (**h**) shows that pyramidal neurons (Pyr) in the DG of the HP are strongly positive for pERK1/2; (**a–d,f–i**) The scale bar = 2 mm; (**e**) The scale bar = 0.5 mm.(**b**) shGnb2l1 icv injection significantly decreased the expression of pCREB protein in the CA1 of HP in acquisition and maintenance. The brown cells represent the pCREB positive cells in the DAB immunostaining. The scale bar = 0.5 mm. (**a,f**) saline + shNC mouse; (**b,g**) saline + shGnb2l1 mouse; (**c,h**) morphine + shNC mouse; (**d,i**) morphine + shGnb2l1 mouse; (**e**) Enlarged, from (**c**) shows that pyramidal neurons (Pyr) in the CA1of the HP are strongly positive for pCREB; (**a–d,f–i**) The scale bar = 2 mm; (**e**) The scale bar = 0.5 mm. (**c**) Representative immunoblot showing the molecular weights of the proteins used to quantify level of ERK1/2 (44/42 kDa), pERK1/2 (43 kDa), CREB (45 kDa) and pCREB (43 kDa) in HP. Signals were normalized to β-actin (43 kDa). The level of ERK1/2 and CREB in HP were not changed after morphine treatment in acquisition and maintenance experiment. An increase in pERK1/2 and pCREB in HP could be detected in both experiments. shGnb2l1icv injection significantly decreased the protein expressions of pERK1/2 and pCREB in HP; (**d**) Comparison of the ratio of the pERK/ERK band between the experimental groups, ^*^p < 0.05 compared with saline + shNC group (n = 5); ^#^p < 0.05 compared with morphine + shNC group (n = 5). (**e**) Comparison of the ratio of the pCREB/CREB band between the experimental groups, ^*^p < 0.05 compared with saline + shNC group (n = 5); ^#^p < 0.05 compared with morphine + shNC group (n = 5).

As shown in Fig. 5a,b, chronic morphine treatment (morphine + shNC) compared to saline administration (saline + shNC) revealed that pERK1/2 and pCREB protein expression were notably up-regulated in the hippocampus. The pyramidal neurons (Pyr) in the CA2 of the hippocampus were strongly positive for pERK1/2 protein in the cytoplasm while pyramidal neurons (Pyr) in the CA1 and dentate gyrus (DG) regions of the hippocampus were strongly positive for pCREB in the nucleus. Under shGnb2l1 treatment (morphine + shGnb2l1), the expression of pERK1/2 and pCREB protein were significantly down-regulated in corresponding areas of the hippocampus, compared to morphine + shNC mice.

Interestingly, in western blotting experiments, there was no significant difference in ERK1/2 and CREB protein expression in the hippocampus during either the acquisition or maintenance experiment. However, notable increases in pERK1/2 and pCREB were observed during acquisition and maintenance of morphine conditioned place preference memory. Two-way ANOVA revealed a difference between the groups (pERK/ERK: F(3, 8) = 34.22, p < 0.0001; pCREB/CREB: F(3, 8) = 16.33, p < 0.001, Fig. 5c–e). The Tukey’s post hoc analysis further showed that compared with morphine + shNC mice, pERK1/2 and pCREB protein expression in the hippocampus were significantly down-regulated in morphine + shGnb2l1 mice during the acquisition (pERK/ERK: q = 5.717, P < 0.05; pCREB/CREB: q = 6.523, P < 0.05) and maintenance experiment (pERK/ERK: q = 9.205, P < 0.05; pCREB/CREB: q = 6.971, P < 0.05). These data collectively suggested that RACK1 activates ERK-CREB signaling and contributes to the acquisition and maintenance of morphine conditioned place preference memory.

## Discussion

In the present study, we investigated the role and mechanism of RACK1 in maintaining morphine conditioned place preference memory. The two main findings of this study are that: (i) persistent morphine conditioned place preference memory was positively correlated with RACK1 in the hippocampus and (ii) that RACK1 promotes maintenance of morphine conditioned place preference memory via activation of an ERK-CREB dependent pathway in hippocampus.

The conditioned-place preference (CPP) paradigm is a widely used animal model to investigate the mechanisms of drug reward memory. Our present data demonstrates that a significant increase in CPP score was induced by repeated morphine administration persisting for 7 days after the end of morphine administration in mice. These results are in line with some recent studies reporting that the morphine induced CPP lasts for about 6 ~ 15 days after cessation of morphine administration in rats^25^,^26^.

The hippocampus is one of the key brain regions involved in long-term memory^6^. Previous studies in our laboratory have demonstrated that RACK1 protein levels are increased in the hippocampus parallel with increased morphine CPP^15^,^16^. Consistently, our present investigation revealed that the maintenance of conditioned place preference memory elicited by morphine is also positively correlated with RACK1 protein level in the hippocampus of mice. Specifically, the administration of morphine and the period occurring 7 days after cessation of morphine administration in mice lead to persistent CPP and produced a long-lasting increase of hippocampal RACK1 levels. In accordance with these findings, we then treated the mice with shGnb2l to interfere with the RACK1 mRNA levels in hippocampus. As predicted, we observed opposing alterations in morphine induced CPP after shGnb2l treatment, both in acquisition and maintenance experiment. In detail, after shGnb2l treatment, the increased CPP score induced by morphine was significantly decreased during acquisition and maintenance experiment. A growing body of evidence has indicated that the persistence of drug-cue memories may be NMDA receptor dependent^27^,^28^,^29^. Since RACK1 plays an important role in regulating NMDAR^9^,^11^, we considered that RACK1 may also be an important player in the promotion and the persistence of a morphine-associated memory and may play a crucial role in drug craving and relapse.

Synaptic structural plasticity is an essential cellular mechanism underlying long-lasting memory^30^, including synapse formation and changes in synaptic associated protein^24^. Synaptophysin (SYP) is a major presynaptic vesicle glycoprotein which is involved in the final steps of exocytosis^31^ and synapse formation in hippocampal neurons^32^. It is well known that chronic morphine treatment induces synaptic plasticity in the nucleus accumbens^33^, hippocampus^8^ and increases expression of SYP^33^,^34^. In agreement with the findings of Heidari *et al.* 2013 and Yong *et al.* 2013, 2014, our present results demonstrated that chronic morphine treatment produces a significant increase in synaptic density and protein levels of the SYP in hippocampal CA1 area in mice, which persisted at least 7 days after the end of morphine injection. Similar to RACK1, SYP protein level in the hippocampus of mice have been also positively correlated with the maintenance of conditioned place preference memory elicited by morphine. Our investigation further demonstrated that shGnb2l icv injection can decrease synaptic density and SYP protein levels in the CA1 area of hippocampus. Altogether, the present results indicate that RACK1 is critical for the maintenance of morphine conditioned place preference memory via hippocampal synaptogenesis.

Extracellular signal-regulated kinases (ERKs) are family members of the mitogen-activated protein kinase (MAPK) pathway, which are strongly associated with long-term synaptic plasticity and learning and memory^35^. Among eight isoforms of ERKs^18^, ERK1 and ERK2 are widely distributed throughout the entire brain and perform an important role in mediating synaptic plasticity and memory formation, especially during exposure to morphine^36^. There is considerable evidence that activation of ERK (pERK1/2) in the hippocampus enhances memory via phosphorylation of the downstream transcription factor CREB on the Ser residue 133^37^. A recent report described that CREB mediated the transcription of SYP by binding phosphorylated CREB (pCREB) on promoters of SYP^22^. The mechanisms underlying the involvement of RACK1 mediated synaptogenesis in the hippocampus during the maintenance and acquisition of morphine CPP require further investigation. Interestingly, the results of our study demonstrated that ERK1/2 and CREB protein levels in hippocampus are not changed, rather pERK1/2 and pCREB protein levels in the hippocampus are up-regulated markedly in both the acquisition and maintenance of morphine conditioned place preference memory. These results are consistent with prior reports that the ERK-CREB pathway is involved in chronic morphine associated memory^38^,^39^, especially in the mechanism for the reconsolidation of drug addiction memory^40^.

Therefore, the above data collectively suggest that changes in ERK and CREB activity may be the underlying mechanisms for the observed hippocampal synaptogenesis which occurs in the maintenance of morphine conditioned place preference memory. Meanwhile, shGnb2l1 icv injection appears to significantly attenuate pERK1/2 and pCREB expression in the hippocampus. Although it is a long way, it someday could be developed into a therapy against CPP or even addiction.

However, there are some limitations in this experiment. The first is the difficult of assessing “addiction” in rodents. The second is the use of the icv injection. In the future study, we will choose intrahippocampal injection to increase the specificity of the treatment.

In summary, our current study suggests that the RACK1 activated ERK-CREB-SYP signaling pathway mediates synaptogenesisin hippocampus and contributes to the acquisition and maintenance of morphine conditioned place preference memory. The findings provide new evidence that RACK1 is a key molecule involved in the maintenance of morphine CPP, and that inhibition of RACK1 can suppress hippocampal synaptogenesis and may prevent the acquisition and maintenance of morphine conditioned place preference memory.

## Methods

### Animals

Male C57BL/6J mice, 6 ~ 8 weeks old weighing 18 ~ 22 g, were obtained from Chengdu Dashuo Experimental Animal Co., Ltd and group-housed in standard conditions with laboratory rodent chow and tap water *ad libitum*. The holding room was maintained under standard conditions of ambient temperature (23 ± 2 °C), humidity (55 ± 5%), and a 12-h light/dark cycle (lights on at 8:00 AM) throughout the whole study.

### Ethics Statement

All animal procedures were performed in accordance with the Committee on the Ethics of Animal Experiments of Sichuan University (Permit Number: 2003-149). All surgery was performed under ether anesthesia, and all efforts were made to minimize animal suffering. All experimental protocols were approved by Sichuan University.

### Drugs

Morphine hydrochloride (1 ml:10 mg, Batch No: 120305, Northeast Pharmaceutical Group Shenyang No. 1 Pharmaceutical Co., Ltd, Shenyang China) was administered intraperitoneally (IP) at a dose of 10 mg/kg.

### The establishment of Gnb2l1 (the gene encoding RACK1) shRNA

The mice Gnb2l1 shRNA were constructed by GenePharma Co. Ltd. (Shanghai, China). The shRNA sequences that target the mice Gnb2l1 sequence (GenBank NM_008143.3) were designed as follows: 5′-CCCACTTCGTTAGTGATGTTG-3′ (PGPU6/GFP/Neo-shGnb2l1). The randomly chosen nonsense sequence 5′-GTTCTCCGAACGTGTACAGT-3′ (pGPU6/GFP/Neo-shNC) was used as a negative control. The sequences of all of the constructs were confirmed by sequencing.

In order to assess the time course requirement of shGnb2l1 to knockdown the RACK1 (Gnb2l1), 5 groups of mice were treated were sacrificed at different time points (6, 12, 18, 24, 48 hours after i.c.v injection of shGnb2l1 (0.3 μg/μl, 5 μl)) and compared with control group which were injected with shNC (0.3 μg/μl, 5 μl). The number of animals in each group was 3. The brains were rapidly removed after the animals were sacrificed and mRNA level of Gnb2l1 was measured by real-time PCR (Fig. S2).

### Conditioned place preference (CPP)

The CPP test was carried out in a two-chamber apparatus (15 cm wide × 30 cm long × 15 cm high) with a sliding partition that divided the main unit into two equal-sized chambers, each of which was enclosed in a sound-attenuating chamber. The two chambers are equipped with different visual and tactile cues: one was white with a textured floor, and the other was black with a smooth floor. When the sliding partition was raised, mice could freely move from one chamber to the other. The partition was closed during training and raised to 7 cm above the floor during habituation and test sessions separated the chambers.

On day 1–3, all animals were habituated to the apparatus by saline i.p. injection followed by access to both chambers for 15 min. The natural preference of the mice (for the white-floor chamber) was scored as the CPP baseline. The mice that showed a strong unconditioned preference (>560 s) for any single compartment were excluded from the study.

From the 4th day on, all mice were engaged in the basic CPP training for ten days. Mice were given morphine (10 mg/kg) or saline i.p. at 10:00 and then confined to the white side of the apparatus for 30 min. On the following day, all of the mice were given saline at 10:00 and then confined to the black section for 30 min. The 2-day procedure was repeated five times (days 4–13). The CPP test was administered under drug-free conditions and performed 24 h after the last conditioning session (day 14). The behavior of the animals was observed and time spent in each compartment was determined by visual analysis of a video recording (15 min). The CPP score was designated as the time spent in the drug-paired compartment on day 14 minus the time spent in the same compartment in the preconditioning phase on day 3 (Test1: acquisition phase). The experimenter scoring the videos of the CPP test was blind to the experimental treatments. 30 min later, half of the mice were sacrificed by decapitation and the hippocampus (HP) was prepared for later analysis.

**Figure 6:**
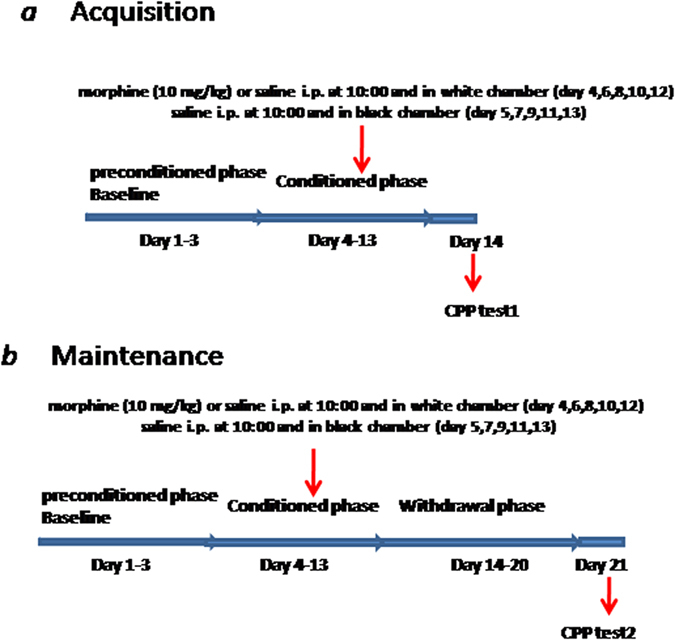
Timeline of experimental procedure. (**a**) Experimental schedule for the acquisition of morphine induced conditioned place preference task; (**b**) Experimental schedule for the maintenance of morphine induced conditioned place preference task.

From days 14 to 20, all the remaining mice were exposed daily to the CPP box with the partition open. Mice received no injections during this time and this phase was marked as the morphine conditioning maintenance phase. On day 21, the maintenance of conditioning was evaluated. The CPP score was designated as the time spent in the drug-paired compartment on day 21 minus the time spent in the same compartment in the preconditioning phase on day 3 (Test 2: maintenance phase). 30 min later, mice were sacrificed by decapitation and the hippocampus (HP) was prepared for later analysis. A timeline for the experiment is shown in Fig. 6. An unconditioned control group that received morphine injections in the home cage and didn’t exposure to the CPP boxes was used to distinguish between the effects of morphine alone and those of learning the association between morphine and the CPP chamber (supplementary information).

### Acquisition experiment

Four groups of mice (n = 10) were trained for morphine-induced CPP. shGnb2l1 or ShNC (0.3 μg/μl, 5 μl) was injected into the i.c.v18 h prior to each morphine administration during conditioning, according to the time course of shGnb2l1 determined to knockdown RACK1(Gnb2l1) (Fig. S2).

### Maintenance experiment

Another four groups of mice (n = 10) underwent a maintenance experiment. Following acquisition of CPP, the mice were given an injection of shGnb2l1 or ShNC (0.3 μg/μl, 5 μl) once every other day in their home cage for another 7 days.

### Hippocampus mRNA and protein preparation

The whole hippocampus (HP) from 5 animals in each conditioned group and unconditioned control group (morphine-exposed, but unconditioned group) was used for Real Time-PCR and Western blot analysis. The tissue was rapidly removed, dissected and stored at −80 °C. The total RNA and protein of HP was extracted by E.Z.N.A.®Total DNA/RNA/Protein Kit (Omega, USA). Protein concentrations were determined using the BCA protein assay kit (Pierce Biotechnology, Rockford, IL).

### Real Time-PCR

Total RNA (1 μg) was subjected to reverse transcription with a Revert Aid First Strand cDNA Synthesis Kit (Thermo scientific Inc, MA, USA). Real-time RT-PCR was performed with CFX96TM Real-Time PCR Detection System, and SYBR@Premix Ex TaqTM II (TliRnaseH Plus) (Takaro Bio Inc, Tokyo, Japan). Primers for Gapdh were used in control reactions. The primer sequences are listed as follows: Gnb2l1 forward primer 5′-GGATCTCAATGAAGGCAAGC-3′, Gnb2l1 reverse primer 5′-TTGCTGCTGGTGCTGATAAC-3′; Gapdh forward primer 5′-GGTTGTCTCCTGCGACTTCA-3′, and Gapdh reverse primer 5′-GGGTGGTCCAGGGTTTCTTA-3′. Reaction mixtures without a cDNA template were used as negative controls. The Ct values were calculated according to the 2^−ΔΔCt^ method.

### Western blot analysis

Equal amounts of proteins (40 μg) were loaded to 12% SDS-PAGE gels and separated by electrophoresis and then transferred to polyvinylidene difluoride (PVDF) membranes (Millipore, Billerica, MA) and incubated separately with mouse monoclonal anti-RACK1 antibody (sc-17754, Santa Cruz Biotechnology, Santa Cruz, CA) (1:1000 dilution), mouse monoclonal anti-CREB antibody (sc-240, Santa Cruz Biotechnology, Santa Cruz, CA) (1:1000 dilution), mouse monoclonal anti-pCREB antibody (sc-81486, Santa Cruz Biotechnology, Santa Cruz, CA) (1:1000 dilution), mouse monoclonal anti-ERK1/2 antibody (sc-135900, Santa Cruz Biotechnology, Santa Cruz, CA) (1:1000 dilution), mouse monoclonal anti-pERK1/2 antibody (sc-81492, Santa Cruz Biotechnology, Santa Cruz, CA) (1:1000 dilution), mouse monoclonal anti-SYP antibody (ab8049, Abcam, Cambridge, UK) (1:1000 dilution) or mouse monoclonal anti-β-actin antibody (TA-09, ZSGB-BIO, Beijing, CHN) as internal control for 2 h at room temperature. Membranes were washed with three times with Tris Buffered Saline with Tween-20 (TBST) and incubated with horseradish peroxidase-conjugated goat anti-rabbit (ZDR-5306, ZSGB-BIO, Beijing, CHN) or goat anti-mouse (ZB-2305, ZSGB-BIO, Beijing, CHN) IgG (1:2000 dilution) for 1 h at room temperature. All proteins were detected using Western Lightning TM Chemiluminescence Reagent Plus and the results were quantitated by scanning densitometry using the Kodak IS4400CF image analysis system and the corresponding software (Eastman Kodak, Rochester, NY).

### Correlational Analysis

Relationships between CPP scores and RACK1, SYP level of HP were assessed using Pearson correlations. We first computed the CPP score for each individual mouse during the following phases: 1) acquisition phase (n = 5); 2) maintenance phase (n = 5) and then correlated the behavioral change with the following corresponding protein expression of HP computed for each individual: 1) RACK1 2) SYP. The Pearson’s r correlation index was also used to ascertain the degree of correlation between the two proteins in hippocampus of mice.

### Brain tissue preparation

Another 5 animals in each conditioned group and unconditioned control group (morphine-exposed, but unconditioned group) were deeply anesthetized with 100 mg/kg of pentobarbital and perfused transcardially with 4% paraformaldehyde/0.6% glutaraldehyde in 0.1M phosphate, pH 7.4. Next the brain was removed immediately and right hemi-brain was fixed in 4% paraformaldehyde for histopathology assay. The hippocampus of the other hemi-brain was fixed in 4% paraformaldehyde, post fixed with 1% osmium tetroxide for electron microscopy assay.

### Electron microscopy (EM)

Coronal sections (0.4 μm) were cut and stained with 1% toluidine blue. All the samples were viewed on transmission electronmicroscope (Zeiss EM900). Five hippocampal sections were analyzed for each animal and the four images were acquired from a randomly selected location within the hippocampus on each slide. All of the synapses in the hippocampal sections were then counted. The total numbers of synapses in the CA1 region from hippocampal sections were counted (a total area of 400 μm^2^ per animal). The numbers of synapses were next measured using Image J software (NIH, Bethesda, MD, USA). Synaptic density was defined as the total numbers of synapses/400 μm^2^. All the data was normalized to saline mice in acquisition phase.

### Brain immunohistochemistry

Coronal sections (4 μm) through the brain were embedded in paraffin and processed for immunohistochemistry. Sections were cleared of paraffin, and endogenous peroxidases were blocked by incubation with 3% H_2_O_2_ and washed. Sections of the brains were then incubated with rabbit serum for 15 min at ambient temperature. Subsequently, the sections were incubated overnight with a goat polyclonal anti-RACK1 antibody (Santa Cruz Biotechnology, Inc., USA, 1:100), anti-pCREB (Santa Cruz Biotechnology, Inc., USA, 1:100), anti-pERK1/2 (Santa Cruz Biotechnology, Inc., USA, 1:100), and anti-SYP (Abcam, Cambridge, UK, 1:100), antibody at 4 °C, followed by the addition of biotinylated rabbit anti-goat IgG secondary antibody (Jinshan, BJ, China). To verify the binding specificity for RACK1, pCREB, pERK1/2 and SYP, some sections were also incubated with primary antibody only (no secondary) or with secondary antibody only (no primary). In these situations, no positive staining was found in the sections, indicating that the immunoreactions were positive in all the experiments carried out. Immunohistochemistry staining was processed in accordance with the manufacturer’s instructions and visualized by the use of diaminobenzidine (DAB) staining. Counterstaining was carried out with Harris hematoxylin (Sigma), according to a procedure from Shroyer *et al.* (2005). Light-microscope images were photographed, and analyzed with Image software (Nikon NIS-Elements D (v 2.30) software) by an observer blind to the treatment groups. Five sections were analyzed for each animal and the four images were acquired from a randomly selected location in each slide. Digital photos were analyzed with Image-pro plus 6.0 (Media Cybernetics, USA) by an observer blind to the treatment groups. Quantification of RACK1, pCREB, pERK1/2 or SYP density per tissue was accomplished by determining the proportion of the area of tissue on each image that was brown (RACK1, pCREB, pERK1/2 or SYP) and blue (remaining tissue) using Image Pro Plus software.

### Statistical analysis

All data are presented as means ± standard error of the mean and statistically evaluated by two-way ANOVA followed by the Bonferroni post hoc test and Tukey’s post-hoc test for between group differences (GraphPad Prism 5, San Diego, California). Linear regression was applied to assess the correlation of two variables by χ^2^ test and the significance of the difference was evaluated by ANOVA using SPSS software v.13.0. The statistical significance was considered as p < 0.05.

## Additional Information

**How to cite this article**: Liu, L. *et al.* RACK1 promotes maintenance of morphine-associated memory via activation of an ERK-CREB dependent pathway in hippocampus. *Sci. Rep.* **6**, 20183; doi: 10.1038/srep20183 (2016).

## Supplementary Material

Supplementary Information
