# Supplementary material for: RACK1 promotes maintenance of morphine-associated memory via activation of an ERK-CREB dependent pathway in hippocampus
Source: Sci Rep. 2016 Feb 2;6:20183. doi: 10.1038/srep20183 (PMC4735742; doi:10.1038/srep20183)
Supplement: Supplementary Information [file srep20183-s1.pdf]

Title

RACK1 promotes maintenance of morphine-associated memory via activation of an ERK-CREB dependent pathway in hippocampus

Category Regular research articles

Authors Litao Liu[1,2], Jiejun Zhu[1,2], Liming Zhou[1,2], Lihong Wan[1,2*]

[1] Department of Pharmacology, West China School of Preclinical and Forensic Medicine, Sichuan University, Chengdu, Sichuan 610041, PR China

[2] Sichuan University "985 project -- Science and Technology Innovation Platform for Novel Drug Development", Sichuan University, Chengdu, Sichuan 610041, PR China

[*]Correspondence should be addressed to

Professor Lihong Wan, Department of Pharmacology, 3-17 Renmin South Road, Preclinical and Forensic Medical College, Sichuan University, Chengdu, Sichuan 610041, P.R. China

E-mail: wanlihong1976@sina.com

Tel: 86-28-85501278

Methods

All mice were randomly assigned to three groups: saline/conditioned unpaired group, morphine/conditioned unpaired group, and morphine/conditioned paired group. The mRNA level of Gnb2l1, protein levels of RACK1 and SYP and synapse density were measured by real-time PCR, Western blotting, immunohistochemistry and EM.

Figure S1. Conditioned paired chronic morphine exposure induced RACK1 high expression and synaptogenesis in hippocampus

(*a*) The mRNA level of RACK1 (Gnb2l1) in hippocampus (HP) was analyzed with real-time PCR, $^{*}p<0.05$ compared with saline group saline/conditioned unpaired group (n=5); (*b*) Representative immunoblot showing the molecular weights of the proteins used to quantify levels of RACK1 (36 kDa) and SYP (38 kDa) in HP. Signals were normalized to β-actin (43 kDa); (*c*) Comparison of the intensity of the RACK1/actin or SYP/actin band between the experimental groups, $^{*}p<0.05$ compared with saline group (n=5); (*d*) Location of RACK1 and SYP protein in CA1 of HP was measured by IHC. (A,D) saline/conditioned unpaired group; (B,E) morphine/conditioned unpaired group; (C,F) morphine/conditioned paired group; The scale bar=0.5 mm; (*e*) Ultrastructural changes in CA1 of HP were observed by TEM, the arrow indicated synapse. (A) saline/conditioned unpaired group; (B) morphine/conditioned unpaired group; (C) morphine/conditioned paired group; Scale bar=20 μM; (*f*) Quantification of synapse density in CA1; data (mean ± SEM) are expressed as percent of synapse density normalized to the appropriate control (the group that received saline injections), $^{*}p<0.05$ compared with saline group (n=5).

Figure S2. The time course of shGnb2l1 to knockdown the Gnb2l1

The mRNA level of RACK1 (Gnb2l1) in hippocampus (HP) during different time point (6, 12, 18, 24, 48 hours after i.c.v injection of shGnb2l1 (0.3 μg/μl, 5 μl)) was analyzed with real-time PCR, $^{*}p<0.05$ compared with shNC group (n=3).

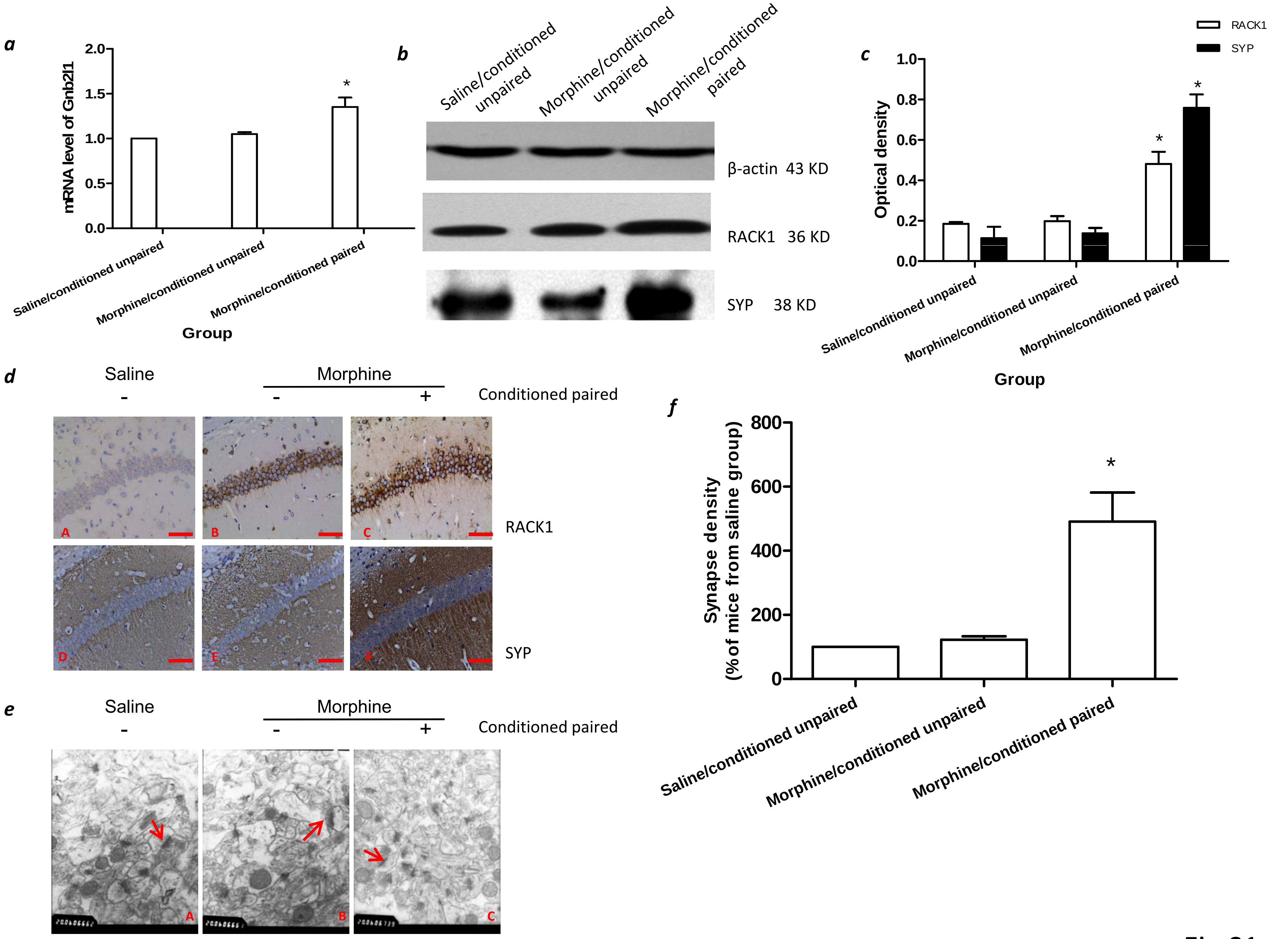

a
mRNA level of Gnb2l1
2.0
1.5
1.0
0.5
0.0
*
Saline/conditioned unpaired
Morphine/conditioned unpaired
Morphine/conditioned paired
Group
b
Saline/conditioned unpaired
Morphine/conditioned unpaired
Morphine/conditioned paired
β-actin 43 KD
RACK1 36 KD
SYP 38 KD
c
RACK1
SYP
Optical density
1.0
0.8
0.6
0.4
0.2
0.0
*
*
Saline/conditioned unpaired
Morphine/conditioned unpaired
Morphine/conditioned paired
Group
d
Saline
Morphine
Conditioned paired
-
-
+
A
B
C
RACK1
D
E
F
SYP
e
Saline
Morphine
Conditioned paired
-
-
+
A
B
C
f
Synapse density
(%of mice from saline group)
800
600
400
200
0
*
Saline/conditioned unpaired
Morphine/conditioned unpaired
Morphine/conditioned paired

Fig S1

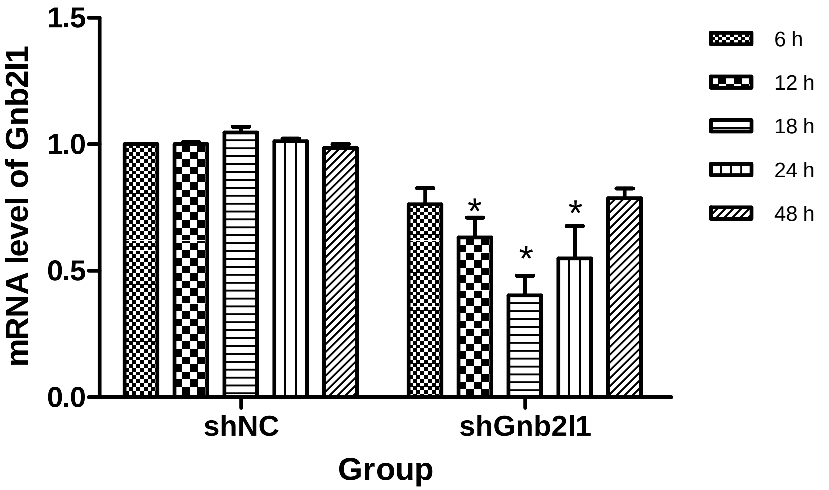

1.5
1.0
0.5
0.0
mRNA level of Gnb2l1
*
*
*
shNC
shGnb2l1
Group
6 h
12 h
18 h
24 h
48 h

Fig S2
